# Acute Stroke due to Electrocution: Uncommon or Unrecognized?

**DOI:** 10.1155/2016/9510863

**Published:** 2016-12-12

**Authors:** Laxmi Kokatnur, Mohan Rudrappa

**Affiliations:** ^1^Louisiana State University Health Sciences Center Shreveport, 1501 Kings Hwy, Shreveport, LA 71104, USA; ^2^Overton Brooks VA Medical Center, 510 E Stoner Ave, Shreveport, LA 71101, USA

## Abstract

The growing dependence on electricity in our daily lives has increased the incidence of electrocution injuries. Although several neurological injuries have been described previously, acute stroke due to electrocution is rare. Our patient, a previously healthy man, was electrocuted after he grabbed a “live” high-voltage wire. Although he was hemodynamically stable, he remained confused with language defects. MRI of the brain showed acute stroke in the bilateral anterior cerebral artery territory and watershed regions of the left middle cerebral artery territory. MR angiogram incidentally showed A1 segment aplasia of the right anterior cerebral artery. Electrocution is known to cause vasospasm leading to end-organ damage similar to that seen in stroke. In our patient, vasospasm of the left anterior circulation likely led to watershed infarcts in the left parietal lobe and bilateral frontal lobes. Due to aplasia of the A1 segment on the right side, perfusion to both frontal lobes was solely from the left anterior cerebral artery.

## 1. Introduction

Electricity played a crucial role in building modern civilization, and humans have paid the price for it. On an average, 411 people will be electrocuted every year [1]. Despite improvements in overall safety measures, this number is likely to increase as we depend more on electricity in every aspect of our lives. The severity of injury depends on the type and strength of current and ranges from a barely perceptible tingling sensation to instant death. Even though several neurological injuries have been described before, acute stroke due to electrocution is only reported in a handful of cases. This underreporting might be due to uncommon occurrence or under recognition. We present a case of acute stroke due to electrocution along with a review of the literature to increase awareness of this rare condition among medical professionals. The early diagnosis and treatment of this condition will have therapeutic implications.

## 2. Case Presentation

**Figure 1:**
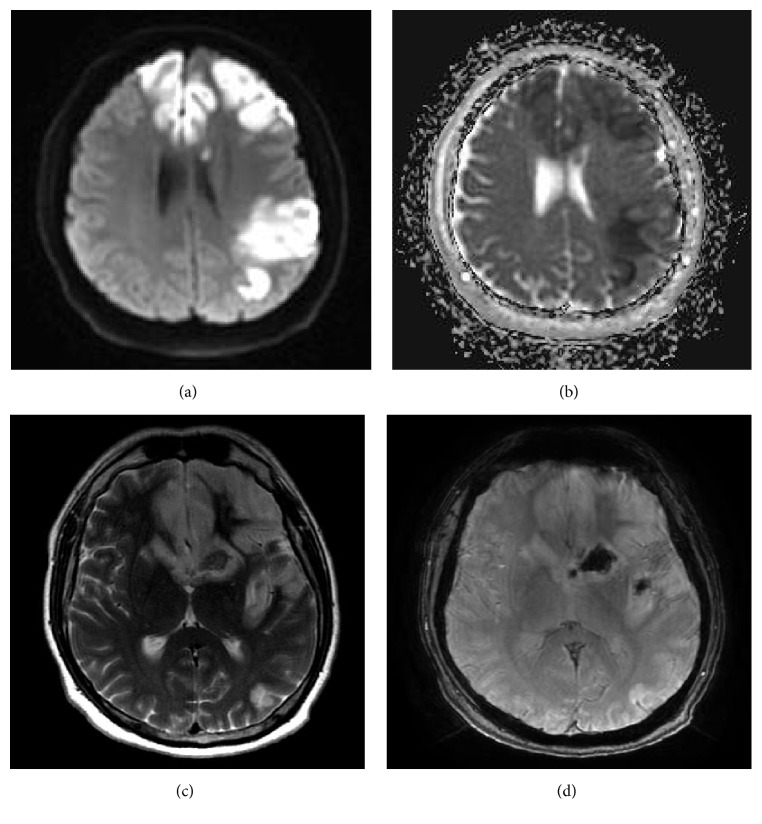
Selected images of MRI of brain. ((a) and (b)) (DWI/ADC) image showing restriction diffusion in bilateral medial frontal lobes and watershed areas in frontal and temporal lobes. (c) T2-weighted image showing hyperintensities in basal ganglia (recurrent artery of Heubner). (d) SWI sequence showing hemorrhagic changes in left basal ganglia and insula.

**Figure 2:**
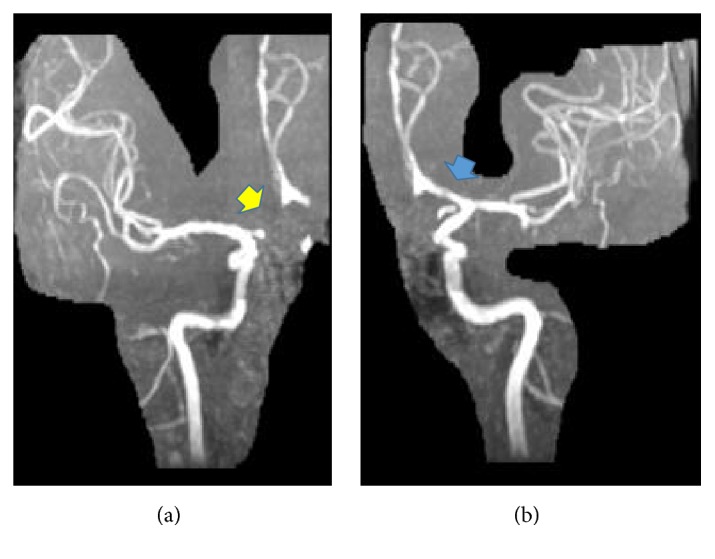
Selected images of MR angiogram. (a) Aplastic A1 segment of right ACA (yellow arrow). (b) Normal A1 segment of left ACA (blue arrow) supplying both frontal lobes.

A 38-year-old white male was found down in the field after he reportedly grabbed a “live” electrical wire. On the way to the local hospital, he regained consciousness but remained confused thereafter. He had sustained second-degree burns at the entry wound in the right palm and had a small exit wound in the right foot. Ecchymosis was also noted on right side of the body over the knee, thigh, and shoulder. A pan CT scan of the body did not reveal any major abnormality, including fractures. He remained hemodynamically stable but was pleasantly confused with a nonfocal neurological examination. A CT scan of the brain showed multiple hypodensities in the left parietal region and frontal region bilaterally. All serology labs were normal except total creatinine kinase levels, which peaked at 1100 U/L. Hence, he was transferred to the burn unit in our hospital for further management of burns with rhabdomyolysis. On examination, he was a pleasant gentleman with complete amnesia of the inciting event. He was alert but disoriented to all spheres. Most of the responses were limited to either head nodding or a few words associated with tangentiality and paraphasia. Repetition was impaired, but comprehension was preserved for simple commands. The cranial nerves and motor and sensory systems were clinically normal except limitation of the right arm's movement due to pain. MRI of the brain using 1.5 Tesla MRI machine showed multiple areas of diffusion restriction in the bilateral medial frontal lobe and bilateral basal ganglia along with watershed areas in the left frontal and temporal regions (Figure 1). Susceptibility-weighted images showed hemorrhage in the left basal ganglia and insula (Figure 1). T2-weighted images showed hyperintensity in the corresponding regions. MR angiogram did not reveal any filling defect or obvious vasospasm but showed aplastic A1 segment of the right anterior cerebral artery (Figure 2). Based on these findings, acute stroke was diagnosed, likely due to vasospasm of the left anterior cerebral circulation due to electrocution. As the patient had presented 3 days after the injury and had shown clinical improvement, it was hypothesized that vasospasm of cerebral vessels might have been resolved when MR angiogram was performed. No hypotension in the field was reported by the first responders and no hypotension was documented during the initial intensive care unit stay at the outside hospital and telemetry monitoring at our center. Also, there was no evidence of end-organ injury due to hypotension induced hypoperfusion arguing against hypotension induced watershed cerebral infarction. Due to aplasia of the right A1 segment, both frontal lobes were supplied by the anterior cerebral artery and vasospasm of the left anterior cerebral circulation might have led to both frontal lobe infarcts along with watershed infarcts of the left middle cerebral artery territory.

An extensive work-up did not reveal any other risk factors for stroke. Transthoracic echocardiogram showed normal structural and functional heart without any intracardiac thrombus; vegetation and bubble study did not show any right to left shunt. Telemetry cardiac monitoring also failed to detect any cardiac arrhythmia. MRA neck did not show any evidence of carotid or vertebral artery disease. The patient's family denied that the patient had any preexisting medical or surgical conditions, including a substance abuse problem. He was an anthropology graduate working as a stunt man for movies and reported being healthy before this event. Despite extensive interviews, the circumstances of the electrocution could not be elucidated, except that he was barefoot at the time of the accident and it was a high-voltage electrical wire. People visiting a local church found him confused on the ground and called for emergency medical services. With supportive treatment, the patient's overall condition improved with some residual language defects. The mild rhabdomyolysis improved after hydration. The entry and exit electric burn wounds responded to local wound care. He was transferred to the neurorehabilitation center for further management and is reported to be recovering well.

## 3. Discussion

Electricity is the flow of electrons through a conductor and is the main source of energy in the 21st century. Voltage is the force responsible for the flow and is measured in volts. Current is the strength of the flow and is measured in amperes. Direct current (DC) flows in only one direction, but alternating current (AC) changes its direction based on the set frequency. Electricity is transported from the production site as AC at a high voltage of 230–700 kV and is gradually reduced to 220–120 V using transformers before it reaches domestic customers. The severity of electrocution injuries depends on the strength of the current and duration of contact. While 1 milliamp (mA) barely causes a tingling sensation, 20 mA can paralyze respiratory muscles. Electrocution with more than 2 amps of current causes significant internal organ and cardiac damage, leading to sudden death [1]. Most electrocution injuries occur in domestic settings with low-voltage current. They rarely require medical attention and are underreported. Injuries from high-voltage electrocution (more than 600 volts) account for 3%–5% of admissions to burn units and 7% of all work-related fatalities [1, 2].

After electrocution, the tissues sustain injuries based on their resistance to the flow of current. The nervous system, blood vessels, and mucous membrane are more prone to injuries as they offer less resistance. Bones, fat, and tendons offer more resistance, but they generate more heat and suffer thermal injury. AC causes tetanic contraction of muscles and the victim is thrown away, breaking the circuit. In contrast, DC of the same strength causes continuous contraction of muscles, making the victim hold on to the source of the current, leading to further electrocution [12, 13]. The nervous system, by virtue of its electrochemical properties, offers the least resistance to the flow of current and hence it is commonly involved in electrocution injuries. Most patients show transient confusion due to alteration of the electrical potential in the brain, which resolves with time. If the brainstem is directly involved, even a low-strength current can cause death due to the involvement of cardiorespiratory centers. Even though most recover from the initial injury, several delayed-onset symptoms of electrocution have also been described [12]. The spectrum of neurological injuries due to electrocution is noted in Table 1.

Acute stroke due to electrocution is uncommon and has been described in only nine cases in the English literature to the best of our knowledge. The salient features of these cases are summarized in Table 2. As the available evidence is from case reports, there is paucity of knowledge regarding the pathophysiology, clinical features, treatment, and outcomes of stroke due to electrocution. Most cases are reported in young males involving low-voltage current. Interestingly, acute stroke can occur even when the central nervous system is not in the direct path of the current, as seen in our patient.

Blood vessels, due to their high water content, can transmit electric current easily to distant sites and cause metastatic injuries. In animal models, direct electrical stimulation of cerebral vessels can cause vasospasm and this effect has been seen at distant sites [14]. In a study looking at vascular injuries due to electrocution in humans, vasospasm was seen in 8 of 12 patients on angiogram [15]. Electrostatic energy in blood vessels can initiate vascular mediopathy and/or intravascular coagulation even when the surrounding tissues appear to be normal [8]. In fact, electricity is used for thrombus generation in animal models when studying carotid artery clots [16]. Acute stroke is also described in electric injury due to lightning [17].

All reported cases were treated as per standard guidelines for the management of acute stroke. Thrombolysis was administered in one patient with no benefit. Later, intra-arterial nimodipine showed a favorable response [10]. Most cases showed clinical improvement, some with complete radiological resolution. The interesting but unfortunate finding of A1 segment aplasia contributed to significant bilateral frontal lobe injury in our patient. A1 segment aplasia is an uncommon developmental defect seen in approximately 2% of cases of stroke on angiograms [18]. Bilateral frontal lobe stroke due to A1 segment aplasia has been reported in only three case reports [19].

## 4. Conclusion

Neurological injuries are common after electrocution injuries. Physicians should be wary of neurological injuries including acute stroke, even when the nervous system does not fall in the path of the current. Timely neuroimaging is helpful and if vasospasm is noted on an angiogram, intra-arterial nimodipine can be considered. The prognosis is excellent with supportive treatment.

## Figures and Tables

**Table 1:** Neurological injuries caused by electrocution.

Type of nervous system	Immediate	Delayed
Central nervous system	Confusion Loss of consciousness Amnesia Acute stroke Seizure Headache Focal brain necrosis Transection of spinal cord Rupture of aneurysm	Transverse myelitis Amyotrophic lateral sclerosis Ascending paralysis Personality changes Delayed brain atrophy

Peripheral nervous system	Nerve palsy	Neuropathies

Autonomic nervous system	Raynaud phenomenon	Complex regional pain syndrome
	Horner's syndrome	
	Keraunoparalysis	

**Table 2:** Published case reports of acute stroke due to electrocution.

Author, year	Age Sex	Voltage	Entry site/exit site	CT head findings	MRI brain findings	Angiogram^*∗*^	Follow-up^*∗∗*^	Other key findings
Singh Jain et al., 2015 [3]	40 Male	High 11000 V	Right arm/axilla	Bilateral, cerebellar, and left occipital hypodensity	Bilateral, cerebellar, and left occipital stroke	Normal	Symptoms improved	18% burns
Bell et al., 2014 [4]	32 Male	High 50000 V Taser gun	NP/NP	Left MCA territory infarct	Left MCA territory ischemic stroke	Distal M1 and proximal M2 left middle cerebral artery filling defect	NP	
Kim et al., 2014 [5]	52 Male	Low 220 V	Right hand/left hand		Left MCA territory ischemic stroke	Focal stenosis of left MCA, left proximal ACA, and proximal basilar artery	Symptoms resolved	Radial nerve neuropathy
Jain et al., 2014 [6]	55 Male	High 66000 V	NP/NP	NP	Left cerebellum ischemia stroke with mass effect	Diffuse narrowing/vasospasm of left vertebral artery	Symptoms improved. Vasospasm resolved at 6 months	
Johl et al., 2012 [7]	43 Male	Low 440 V	Scalp/left foot	NP	Bilateral medullary pyramids and pons	NP	Symptoms improved. MRI changes resolved	Spinal card infarction
Chen et al., 2012 [8]	62 Female	Low 110 V	Left hand/NP	NP	Left paramedian pons ischemic stroke	Narrowing of proximal basilar artery, bilateral distal vertebral artery, and MCA likely due to thrombosis	Symptoms resolved. Stenosis progressed	Protein C deficiency
Verma et al., 2014 [9]	30 Male	Low 240 V	NP/NP	Right MCA infarct with mass effect	NP	NP	Symptoms improved. Mass effect resolved	Acute myocardial infarction
Huan-Jui et al., 2010 [10]	50 Male	Low 110 V	Both hands/NP	Normal	Right frontotemporal area, basal ganglia, and corona radiate stroke	Segmental narrowing of siphon of right internal carotid artery and M1 segment of middle cerebral artery	Symptoms did not improve. Vasospasm resolved	TPA was given. Vasospasm improved with intra-arterial nimodipine
Kamyar and Trob, 2009 [11]	28 Male	Low 220 V	Both hands/left foot	Normal	Mesial occipital bilateral infarction	NP	Symptoms improved	Cardiac arrest for 10 min. Cardiogenic-ischemic encephalopathy

NP: not reported. *∗* refers to either CT MR or digital subtraction angiogram. *∗∗* indicates that most cases report short follow-up period.
